# Biomolecular condensates: insights into early and late steps of the HIV-1 replication cycle

**DOI:** 10.1186/s12977-023-00619-6

**Published:** 2023-04-07

**Authors:** Francesca Di Nunzio, Vladimir N. Uversky, Andrew J. Mouland

**Affiliations:** 1 Advanced Molecular Virology Unit, Department of Virology, Institut Pasteur, Université Paris Cité, 75015 Paris, France; 2 Department of Molecular Medicine and USF Health Byrd Alzheimer’s Research Institute, Morsani College of Medicine, University of South Florida, Tampa, FL 33612 USA; 3 Lady Davis Institute at the Jewish General Hospital, Montréal, QC H3T 1E2 Canada; 4 Department of Microbiology and Immunology, McGill University, Montréal, QC H3A 2B4 Canada; 5 Department of Medicine, McGill University, Montréal, QC H4A 3J1 Canada

**Keywords:** Retrovirus, HIV-1, Liquid–liquid phase separation (LLPS), Biomolecular condensates (BMC), Membraneless organelles (MLO), Intrinsically disordered protein (IDP), Droplet-promoting region, Intrinsically disordered region (IDR), Nuclear pore complex, Integration, Transcription, Virus assembly, Protein–protein interactions, Coacervation, Posttranslational modification, Molecular recognition feature

## Abstract

A rapidly evolving understanding of phase separation in the biological and physical sciences has led to the redefining of virus-engineered replication compartments in many viruses with RNA genomes. Condensation of viral, host and genomic and subgenomic RNAs can take place to evade the innate immunity response and to help viral replication. Divergent viruses prompt liquid–liquid phase separation (LLPS) to invade the host cell. During HIV replication there are several steps involving LLPS. In this review, we characterize the ability of individual viral and host partners that assemble into biomolecular condensates (BMCs). Of note, bioinformatic analyses predict models of phase separation in line with several published observations. Importantly, viral BMCs contribute to function in key steps retroviral replication. For example, reverse transcription takes place within nuclear BMCs, called HIV-MLOs while during late replication steps, retroviral nucleocapsid acts as a driver or scaffold to recruit client viral components to aid the assembly of progeny virions. Overall, LLPS during viral infections represents a newly described biological event now appreciated in the virology field, that can also be considered as an alternative pharmacological target to current drug therapies especially when viruses become resistant to antiviral treatment.

## Background

Liquid–liquid phase separation (LLPS) relies on the condensation of proteins and often (but not always) RNAs or DNAs (or other biological polymers) into membraneless organelles (MLOs), also known as biomolecular condensates (BMCs), allowing select molecules to become concentrated while excluding others. This process, which represents “coacervation”, if it is driven by the polyelectrostatic interactions between macromolecules undergoing LLPS, compartmentalizes fundamental processes in the nucleus and in the cytoplasm. One should keep in mind that coacervation is one of several types of LLPS which occurs in solutions of oppositely charged macromolecular species including proteins, nucleic acids, polymers, and colloids. The other mechanisms driving LLPS include weak pi-pi, cation-pi, and hydrophobic interactions [1]. Compartmentalization of function relies on MLOs such as the nucleolus, nuclear speckle, neuronal RNA trafficking granules, stress granules, and processing bodies [2, 3] enables key gene expression steps in which multiple components must come into proximity for function [4–15]. The presence of intrinsically disordered regions (IDR) in proteins and multivalent macromolecular interactions between proteins or between proteins and nucleic acids, lead to liquid demixing and phase separation [16–22]. One of the theories describing this process in the cell is the “stickers-and-spacers” model [23] which considers protein and RNA as flexible polymers where sequence motifs, the “stickers,” determine attractive interactions between different molecules while other regions act as inert “spacers” between them. Stickers on the protein/RNA chain, once a critical concentration threshold is reached, can induce a separation into a denser phase that coexists with a more dilute phase.

It is commonly accepted that RNA played a key role in the evolution of life. In the “RNA world” theory, the primitive RNA arose spontaneously using specific features of the early Earth environment. RNA can serve as template to code for proteins. Experiments in vitro show that RNA molecules perform a wide range of catalytic reactions. Furthermore, RNA and proteins carrying IDRs can assemble into the biocondensates, which are formed by LLPS. These coacervates have been implicated in the origins of life, acting as bioreactors, where nucleotides, magnesium, and RNA molecules accumulate and increase in local concentration [24, 25]. Indeed, one of the most interesting questions in science is how life first appeared. In 1938, the Soviet biochemist Alexander Oparin in “The Origin of Life” proposed that life originated as coacervate drops of organic materials [26]. His theory was based on simple observation that droplets of organic molecules merge spontaneously from an otherwise diluted solution. The inability to demonstrate the presence of membrane barriers accounted for the loss of credibility in Oparin’s coacervate idea. Although in recent years this theory has been reexamined due to the discovery of numerous intracellular, membraneless BMCs [27]. Such liquid droplets or MLOs are generated by LLPS. LLPS ensures the tightly controlled performance of complex cellular functions in a limited space and in a spatiotemporal manner. LLPS is based on multivalent interactions among IDRs and/or modular interacting domains of some components [5, 28, 29]. LLPS is a widespread phenomenon that can explain numerous cellular functions [30–32], illness states [33], and host-pathogens coexistence [34]. It is not surprising that viruses learned to usurp LLPS [35]. “Viral factories” and other “viral replication centers” in infected cells are central to virus replication and are thus defined as BMCs [36–40].

The oligomerization of IDR-rich proteins is key to driving phase separation. Furthermore, there are several examples in nature, such as nascent ribosomal RNA (rRNA) transcripts [41, 42] or long non-coding RNA (lncRNA) such as Neat1 [43] and other types of RNAs are associated with specific DNA loci that may act as scaffolds for locally enriching self-interacting IDPs [44]. Several proteins appear to self-oligomerize leading to phase separation, like NPM1 that pentamerizes to form a radial array of IDRs and RNA-binding domains important for phase separation and nucleolus assembly [30, 45]. The oligomerization of the protein G3BP1 is also important for stress granule condensation [46]. It is also clear that the LLPS-driven biogenesis of BMCs can be dysregulated by pathological gene mutations [47] that lead to neurodegenerative disease [48–50] and to tumorigenesis [48–50].

The importance of LLPS and related BMCs/MLOs in relation to infectious diseases has recently gained widespread attention due in part to focussed research efforts on the RNA virus, SARS-CoV-2, etiologic to COVID-19. SARS-CoV-2 expresses a (N)nucleocapsid protein that phase separates and co-condenses with several host and viral proteins in the assembly of its RNA genome to aid viral packaging [11, 51, 52]. Indeed, N acts as a filter to select vRNA full-length genome excluding subgenomic vRNA from BMCs. Likewise, HIV-1 and other retroviral proteins possess intrinsically disordered domains that are juxtaposed with RNA- and Zn-binding domains (RBD and ZnF) [11, 53, 54] that promote protein co-condensation and confer liquid-like behaviour of resulting condensates [55]. HIV-1 Gag BMCs exhibit fluid-like behaviour characterized by fluid movement, fusion and fission [56]. The fluidity of BMCs can also be enhanced by RNA thereby promoting more dynamic interactions [57, 58], likely playing a role for viruses with RNA genomes. Endogenous retroviruses (ERV) also elicit BMC assemblies for replication [59, 60]. A recent study indicates that ERV retrotransposons have the ability to hijack biomolecular condensates enriched of transcriptional regulatory proteins in pluripotent cells. ERVs reactivation seems to compete with super-enhancers for transcriptional condensates in pluripotent cells. Thus, the repression of ERVs is important for the correct cellular fates This mechanism explains why thousands of transposition-incapable ERVs are repressed in mammals [60].

## Retroviral replication: implications for membraneless organelles generated by phase separation

Fifty-two years ago, the discovery of reverse transcription [61, 62] amended the central dogma showing that not only the DNA but also the RNA can contain the genetic information to be coded into protein through a reverse transcription process that converts the RNA in DNA, the process, which is the hallmark characteristic retroviruses. The discovery of reverse transcription links RNA to the DNA world. Before the discovery of reverse transcription, the first evidence of the existence of retroviruses had been obtained from Robin Weiss who was able to demonstrate that Env protein is expressed from a retroviral genome integrated in the host DNA [63].

**Fig. 1:**
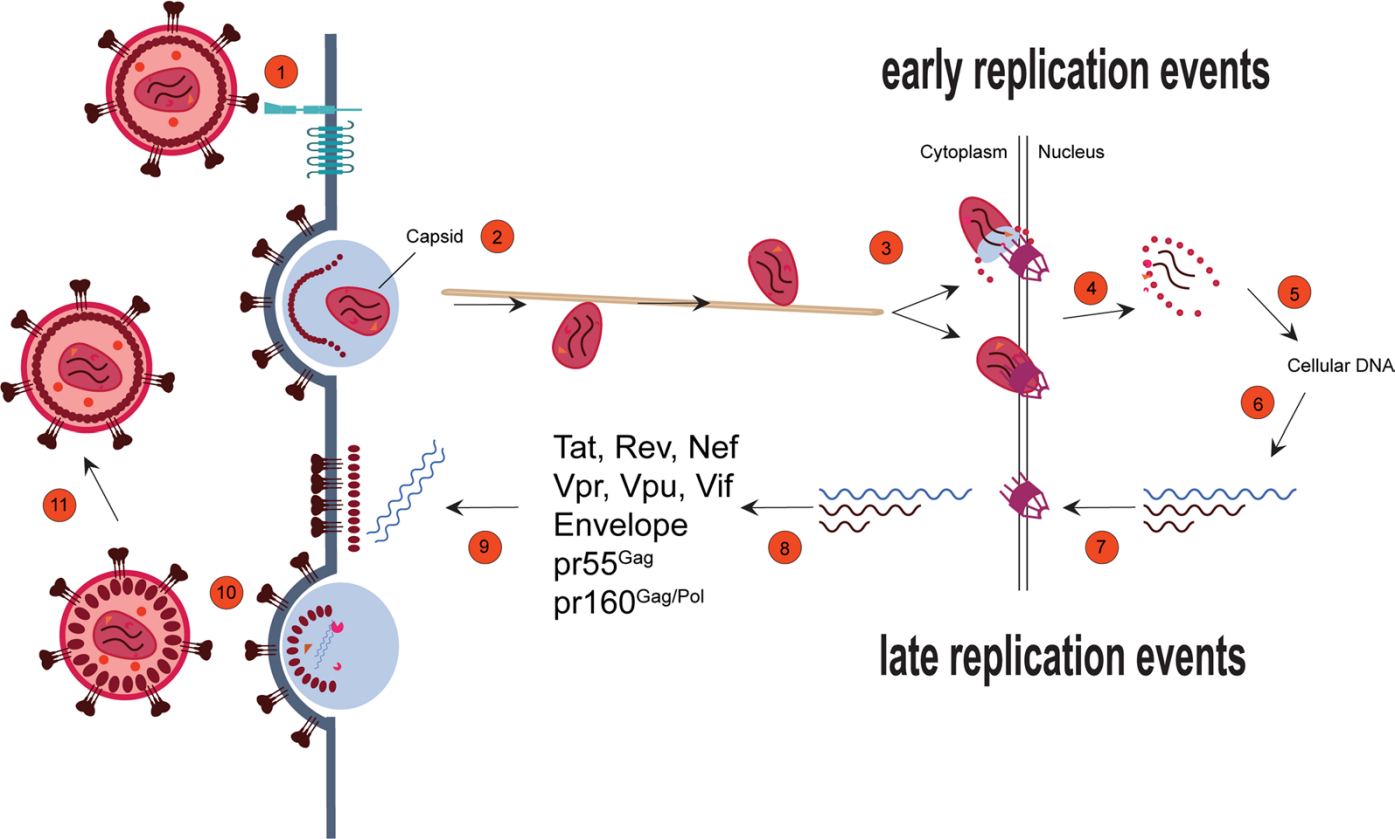
Evolving and potential roles for biomolecular condensates at various steps of the retroviral replication cycle. 1, engagement with host cell receptors and viral entry; 2, membrane fusion and intracellular trafficking of the viral capsid; 3, nuclear entry; 4, uncoating and completion of reverse transcription; 5, integration into host DNA; 6, transcription, intranuclear trafficking of the viral ribonucleoprotein; 7, nucleocytoplasmic mRNA export through the nuclear pore with completely spliced mRNAs exported first then the singly-spliced and unspliced mRNAs; 8, mRNA engagement with the host translation machinery to generate early (Tat, Rev and Nef) and late (pr55^Gag^, pr160^Gag/Pol^, Vpr, Vpu and Vif) viral gene products, as indicated, and genomic RNA selection; 9, genomic RNA and viral protein trafficking to virus assembly sites; 10, virus budding; and 11, virus maturation mediated by the viral protease substrates, pr55^Gag^ and pr160^Gag/Pol^. Please see text for details on condensation at virus replication steps

Retroviruses are a large family of RNA viruses, and they are classified in orthovirinae and spumavirinae, the former with six genera, the latter with five [64]. Among the family of orthoretrovirinae, there is the subfamily of lentiviruses that has the ability to infect both dividing and non-dividing cells. Early and late phases define the retroviral replication cycle (Fig. 1). The early steps begin with viral fusion to the cellular membrane followed by the cytoplasmic viral journey of the viral core to reach the host chromatin for the viral integration into the host genome. As a result, the host cell becomes a chimera, and the host transcriptional mechanism takes over the transcription of viral genes in a similar way to its own genes, which must be highly expressed. Viral gene transcription, nuclear export of viral RNAs, mRNA translation and the assembly of new viral particles represent key late steps of the retroviral replication cycle.

Condensates are formed during both the early and late viral cycle phases to create microenvironments to facilitate and optimize viral replication and likely, to shield the viral genome from anti-viral innate immune host responses (Fig. 1). Often, condensates serve to organize cellular biochemistry by bringing the correct partners together in a confined environment to accelerate reaction rates, to quickly reply to stress, or to act as filters. These are new biological phenomena that have only recently been recognized as a part of key steps in the replication cycle of the HIV-1, etiological to the AIDS pandemic. Certain proteins function as scaffolds and are critical elements involved in driving the biogenesis of condensates (drivers or scaffolds), as it is the case for host proteins, SRRM2 and SON, that are essential for the formation of nuclear speckles, which are also considered MLOs [65, 66]. The implications for MLOs in retroviral replication based on recent literature and bioinformatic analyses are discussed below.

### Early events of the retroviral replication cycle

#### Nuclear translocation

**Fig. 2:**
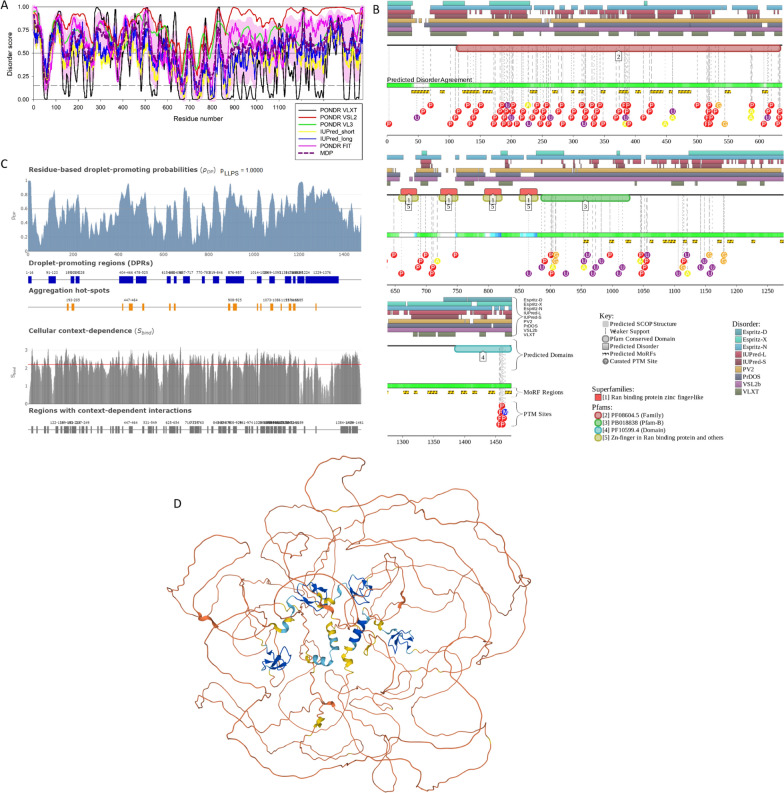
Functional disorder analysis of human Nup153 protein (UniProt ID: P49790). **A** Per-residue disorder profile generated by RIDAO [138]. Per-residue intrinsic disorder profiles generated by PONDR^®^ VLXT, PONDR^®^ VL3, PONDR^®^ VSL2, PONDR^®^ FIT, IUPred_long and IUPred_short. Mean disorder profile (MDP) is calculated by averaging of the disorder profiles of individual predictors. Light pink shade represents MDP error distribution. The thin black line at the disorder score of 0.5 is the threshold between order and disorder, where residues/regions above 0.5 are disordered and residues/regions below 0.5 are ordered. The thin dashed line at the disorder score of 0.15 is the threshold between order and flexibility, where residues/regions above 0.15 are flexible and residues/regions below 0.15 are highly ordered. **B** Functional disorder profile generated by the D^2^P^2^ platform [139]. Here, the IDR localization predicted by IUPred, PONDR^®^ VLXT, PONDR^®^ VSL2, PrDOS, PV2, and ESpritz are shown by 9 differently colored bars on the top of the plot, whereas the blue-green-white bar in the middle of the plots shows the agreement between the outputs of these disorder predictors, with disordered regions by consensus being shown by blue and green. The two lines with colored and numbered bars above the disorder consensus bar show the positions of functional SCOP domains [140, 141] predicted using the SUPERFAMILY predictor [142]. Positions of the predicted disorder-based binding sites (MoRF regions) identified by the ANCHOR algorithm are shown by yellow zigzagged bars [143]. Locations of the sites of different PTMs identified by the PhosphoSitePlus platform [144] are shown at the bottom of the plot by the differently colored circles. Here, phosphorylation, ubiquitylation, acetylation, glycosylation and methylation sites are shown by red, violet, yellow, orange and blue circles with the letters P, U, A, G, and M, respectively. An interactive profile generated for human Nup153 protein by D^2^P^2^ can be found at the following link: https://d2p2.pro/view/sequence/up/P49790. **C** Evaluation of LLPS predisposition and existence of regions with context-dependent disorder [145]. Top plot represents sequence distribution of the probability of forming a droplet state through liquid–liquid phase separation (p_LLPS_). Proteins with p_LLPS_ ≥ 0.60 are droplet-drivers, which can spontaneously undergo liquid–liquid phase separation. Droplet-client proteins have p_LLPS_ < 0.60, but possess droplet-promoting regions (DPRs), which can induce their partitioning into condensates. Positions of DPRs (which contain residues with p_DP_ ≥ 0.60 capable to promote liquid–liquid phase separation) are shown by blue bar. Positions of aggregation hot-spots that drive aggregation of condensates are displayed by orange boxes. These regions are droplet-promoting (p_DP_ ≥ 0.60) and exhibit high interaction mode divergence (S_BIND_ ≥ 2.2) making them sensitive to the cellular context. The bottom plot shows sequence distribution of the S_BIND_ values that characterize the ability of residues to switch between different binding modes. These are computed as Shannon entropy values of the binding mode distribution [146]. Plot also shows regions with context-dependent interactions that change protein interaction behavior and binding modes under different cellular conditions. These residues can be ordered or disordered with S_BIND_ ≥ 2.25. **D** Model of the CPSF6 3D structure generated by AlphaFold [147]. Structure is colored based on the AlphaFold-generated per-residue confidence score (predicted local distance difference test, pLDDT) values, where orange, yellow, cyan, and blue colors correspond to the segments predicted by AlphaFold with very high very low (pLDDT < 50), low (70 > pLDDT > 50), high (90 > pLDDT > 70), and (pLDDT > 90) confidence

The nuclear pore complex (NPC) is the path used by HIV-1 to enter the nucleus of both dividing and non-dividing cells**.** The viral capsid docks at the NPC through its interaction with the cytoplasmic filaments of the pore, mainly formed by the nucleoporin RanBP2 [67, 68] and next, translocates through the NPC channel helped by an intranuclear nucleoporin the Nup153 [69, 70]). Thus, the viral capsid is the determinant responsible for the viral nuclear entry because it interacts with specific nucleoporins to first dock at the NPC and then translocate through it [67–71]. Once inside the nucleus, the virus will enter in contact with host nuclear factors as well as with an euchromatin environment located all around the nuclear basket of the NPC. The nuclear basket of the NPC is surrounded by euchromatin [71, 72], contrary to the chromatin under the nuclear envelope, which is heterochromatin, known as lamin associated domains (LADs). HIV-1 favors integration in active chromatin, especially in the chromatin near the NPC [72, 73]) and others near nuclear speckles [74, 75]. On the other hand, HIV-1 disfavors integration in LADs that are silent chromatin regions [70, 74]. How does the partitioning between euchromatin and heterochromatin underneath the NE occur? Some nucleoporins, especially the ones carrying IDRs, like Nup153 that has phenylalanine-glycine (FG) domains could assist in forming phase-separated transcriptional compartments [76]. Perhaps the proximity to the nuclear pore and the abundance of proteins with IDRs at super-enhancers could recruit Nups, which could assist in phase separation through mediating proximity to nuclear speckles (NSs), which are surrounded by active chromatin, and NPCs. Nup153 is an important component of the nuclear basket and it is also part of a free intranuclear population and holds 29 FG repeats that are highly disordered [77]. Figure 2 and Table 1 represent results of functional disorder analysis of human Nup153 and shows that this host protein contains high levels of intrinsic disorder regions as evidenced by the per-residue disorder profile generated by a set of commonly used disorder predictors PONDR^®^ VLXT, PONDR^®^ VL3, PONDR^®^ VSL2, PONDR^®^ FIT, IUPred_long and IUPred_short) (Fig. 2A) and a functional disorder profile generated by the D^2^P^2^ platform (Fig. 2B). Therefore, both analyses show that most of the protein is expected to be disordered. Furthermore, Fig. 2B shows that Nup153 contains multiple disorder-based binding sites (i.e., IDRs that are capable of folding at interaction with specific binding partners and known as molecular recognition features, MoRFs). Interestingly, Nup153 has been found to mediate chromatin structure and influence transcription by modulating CTCF and cohesin binding across *cis*-regulatory elements and topologically associating domains (TAD) boundaries [78]. Note that MoRFs and post-translational modifications (PTMs) are distributed throughout the entire sequence of this protein. Figure 2C and Table 1 suggest that Nup153 has a very strong propensity to phase separate (its LLPS propensity evaluated by FuzDrop is equal to 1, and possesses numerous droplet-promoting regions (DPRs), in line with Nup153 gel formation properties [79]. This indicates that Nup153 can serve as a driver (or a scaffold) controlling the formation of Nup153-containing BMCs. Finally, Fig. 2D provides an overview of the expected 3D structure of this protein generated by AlphaFold and shows that a very significant part of Nup153 is highly disordered.

**Table 1:** Levels of intrinsic disorder and LLPS predispositions of host and viral proteins related to HIV-1 replication cycle

	PPIDR_PONDR VSL2_ (%)	P_LLPS_
Viral proteins		
CA	22.94	0.1607
pr55^Gag^	43.40	0.7147
RT	19.11	0.1892
IN	17.79	0.1681
NC	100.00	0.6502
Host proteins		
Nup153	94.64	1.0000
CPSF6	79.31	0.9976
LEDGF	77.74	0.9963

#### Viral post-nuclear entry steps

**Fig. 3:**
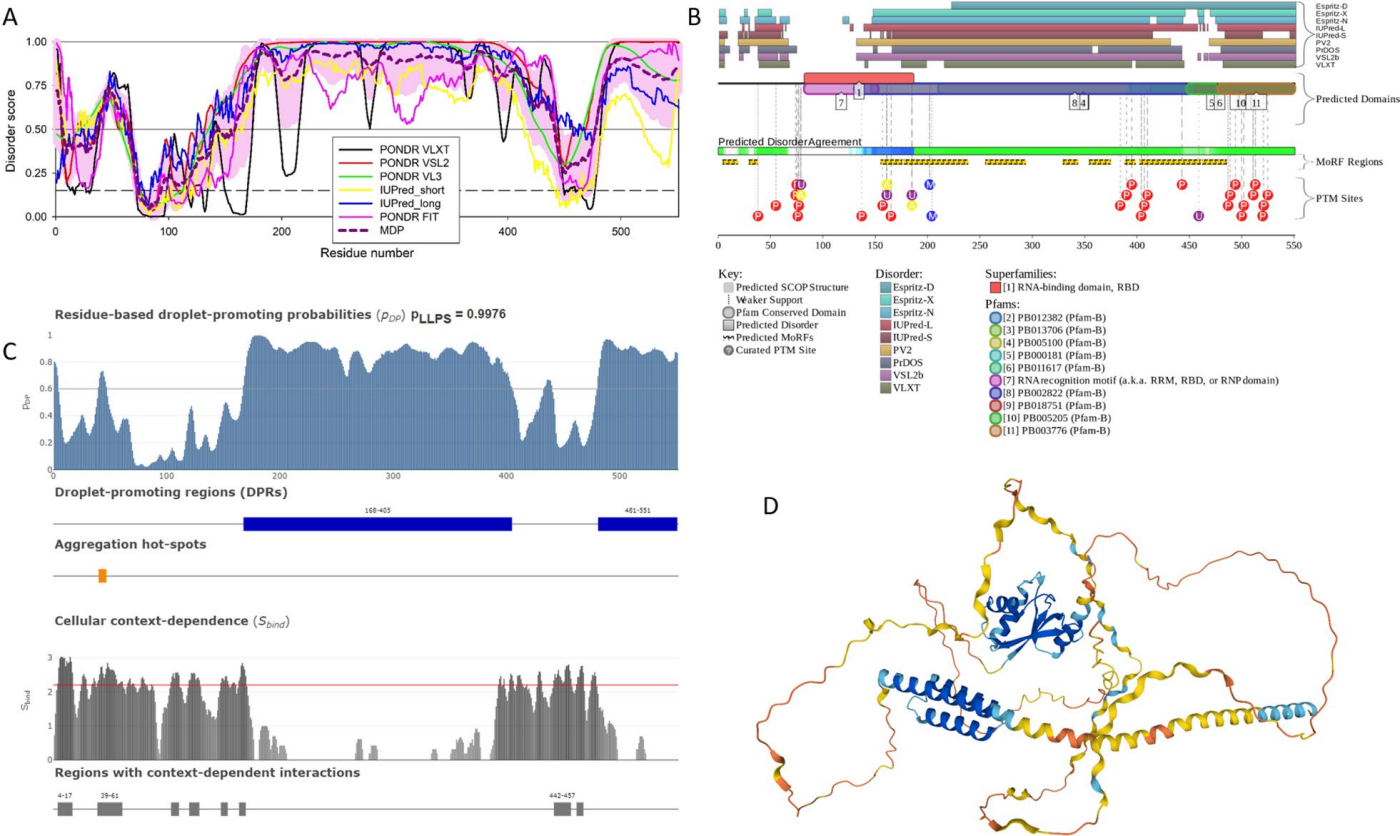
Functional disorder analysis of human CPSF6 protein (UniProt ID: Q16630). **A** Per-residue disorder profile generated by RIDAO [138]. **B** Functional disorder profile generated by the D^2^P^2^ platform [139]. Here, phosphorylation, ubiquitylation, acetylation, and methylation sites are shown by red, violet, yellow, and blue circles with the letters P, U, A, and M, respectively. A D^2^P^2^-generated interactive profile of the human CPSF6 protein can be found at the following link: https://d2p2.pro/view/sequence/up/Q16630. **C** Evaluation of LLPS predisposition and existence of regions with context-dependent disorder [145]. **D** Model of the CPSF6 3D structure generated by AlphaFold [147]

Since the formation of various MLOs represents one cellular stress response mechanism, it is not surprising to find that HIV-1 infection prompts the formation of MLOs. These intranuclear viral-host structures defined as HIV-1 MLOs [75] characterize viral post-nuclear entry steps. In these organelles, the scaffold protein is the cleavage and polyadenylation specific factor 6 (CPSF6) that contains mixed-charge domains (MCDs), which are protein domains or regions/clusters characterized by high enrichment for positive and negatively charged amino acids, where the relative numbers of basic to acidic components may be balanced or skewed [80–83], at the C-terminus region of the protein. MCDs seem to be critical for its capacity to phase separate [82]. CPSF6 binds the hydrophobic pockets of the viral core [84, 85]. Interestingly, the human CPSF6 is characterized by high levels of intrinsic disorder (see Fig. 3A, B and Table 1) and contains multiple disorder-based binding sites (i.e., disordered regions capable of disorder-to-order transition at interaction with specific partners) and multiple sites of various PTMs (see Fig. 3B). Furthermore, this protein shows high LLPS propensity (p_LLPS_ = 0.9976) and contains two long droplet-promoting regions and multiple regions with context-dependent interactions (see Fig. 3C). These observations indicate that CPSF6 can serve as a driver of LLPS (see Table 1). The fact that CPSF6 is a highly disordered protein is further supported by Fig. 3D representing the 3D structure of this protein generated by AlphaFold. Note that the most structured regions of this protein that are predicted with highest confidence are the RNA-binding motif (RRM) domain (residues 81–161) and the segment required for the RNA binding (residues 405–551) [86] (regions in blue and in cyan).

**Fig. 4:**
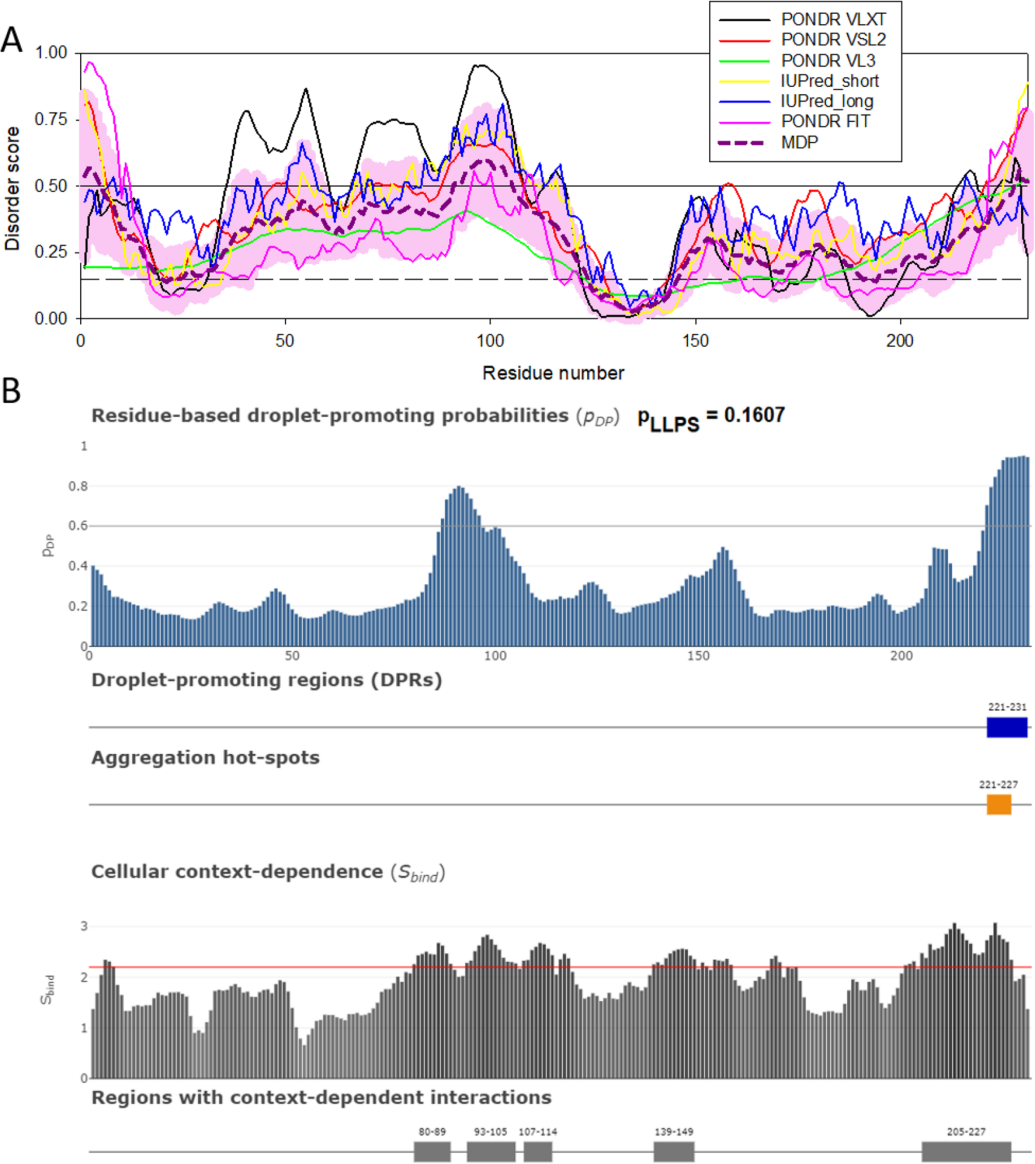
Disorder analysis of the HIV-1 capsid protein (UniProt ID: P12493; residues 133–363). **A** Per-residue disorder profile generated by RIDAO [138]. **B** Evaluation of LLPS predisposition and existence of regions with context-dependent disorder [145]

The presence of several IDRs and DPRs can account in part for the formation of HIV-1 MLOs in combination with the presence of the viral capsid in the nucleus [75]. It is possible that CPSF6 condensates act as a scaffold to recruit the viral capsid as a client. Of note, client molecules are not able to form condensates by their own, but through direct interaction with scaffolds can associate [5, 87] with condensates [87]. In line with these considerations, Fig. 4 and Table 1 show that although capsid is predicted to contain noticeable levels of disordered and flexible regions and possesses five regions with context-dependent interactions, this protein is incapable of driving LLPS. However, although being characterized by a low p_LLPS_ value of 0.1607 (see Fig. 4B, and Table 1), capsid is predicted by FuzDrop to contain one DPR. These observations suggest that the capsid can serve as an MLO client, which is in line with experimental data [92]. It is possible that the DPR could be exposed when the fragmented viral capsid while transiting inside the nucleus [88, 89], another possibility is that the fragments of the viral capsid or CA monomers formed after viral uncoating can have a role in maintaining the HIV-MLOs once established in the nucleus.

#### Reverse transcription

**Fig. 5:**
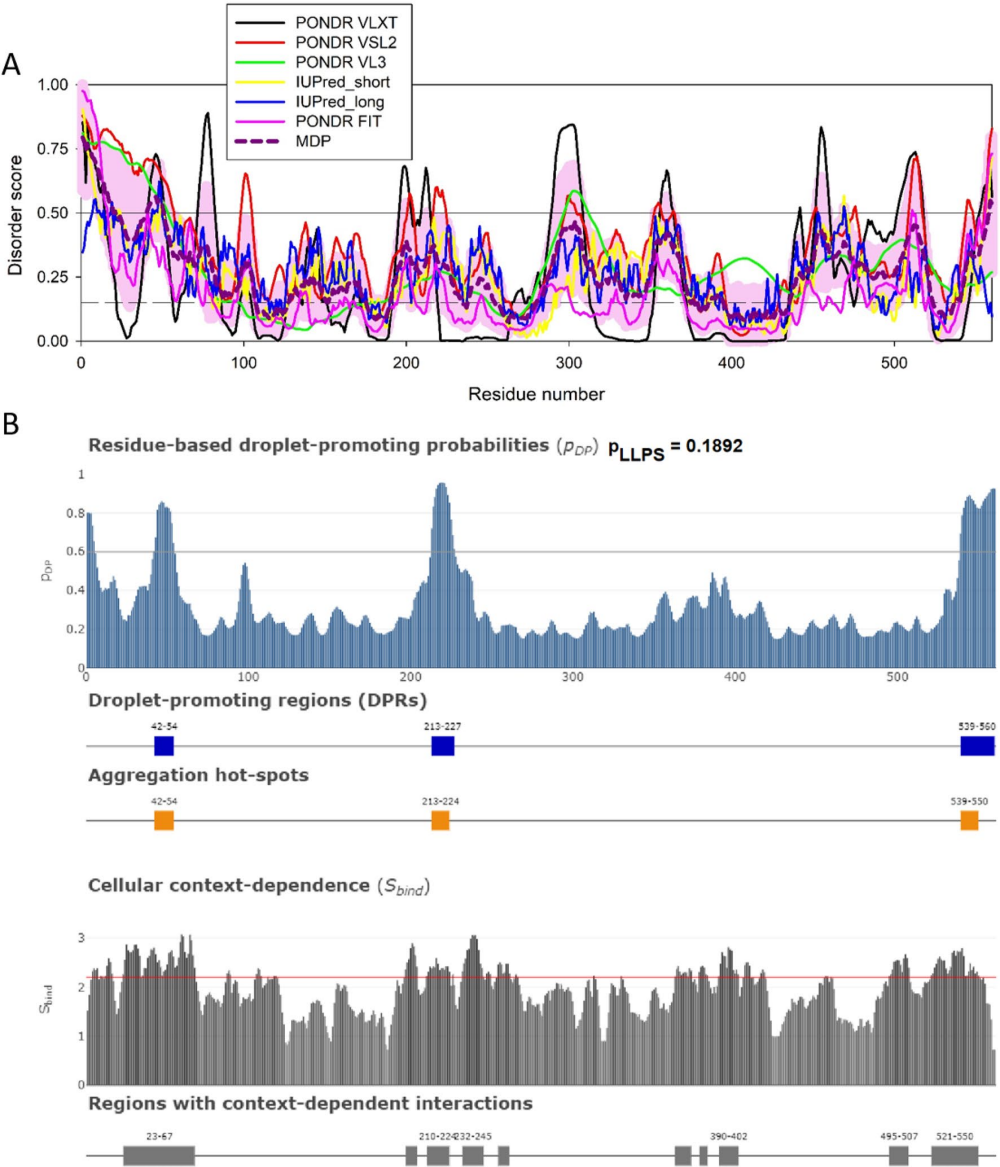
Disorder analysis of the HIV-1 reverse transcriptase (UniProt ID: P12497; residues: 588–1147). **A** Per-residue disorder profile generated by RIDAO [138]. **B** Evaluation of LLPS predisposition and existence of regions with context-dependent disorder [145]

HIV-1 MLOs contain the viral RNA genome that is reverse transcribed in these organelles to generate the viral DNA [75, 90] the only viral form capable of integration into the host chromatin. Intriguingly, HIV-1 MLOs could act as bioreactor to preserve viral cores intact that represent a confined space where the reverse transcription has been shown to occur in vitro [91] or to keep multiple reverse transcription (p66/p51) heterodimers together, likely, acting in the context of a condensate. Another possibility is that HIV-1 MLOs keep multiple cores together and that the reverse transcription occurs within them. Running bioinformatics analysis, we observed that the reverse transcriptase enzyme is expected to contain several IDRs (Fig. 5A) and possesses three droplet-promoting regions (Fig. 5B). However, the overall droplet-promoting probability of this protein is low (below the threshold of 0.6, see Table 1). Therefore, these observations suggest that reverse transcriptase has a low probability to induce LLPS but likely serves as a client of liquid droplets, as shown recently [92].

#### Integration

**Fig. 6:**
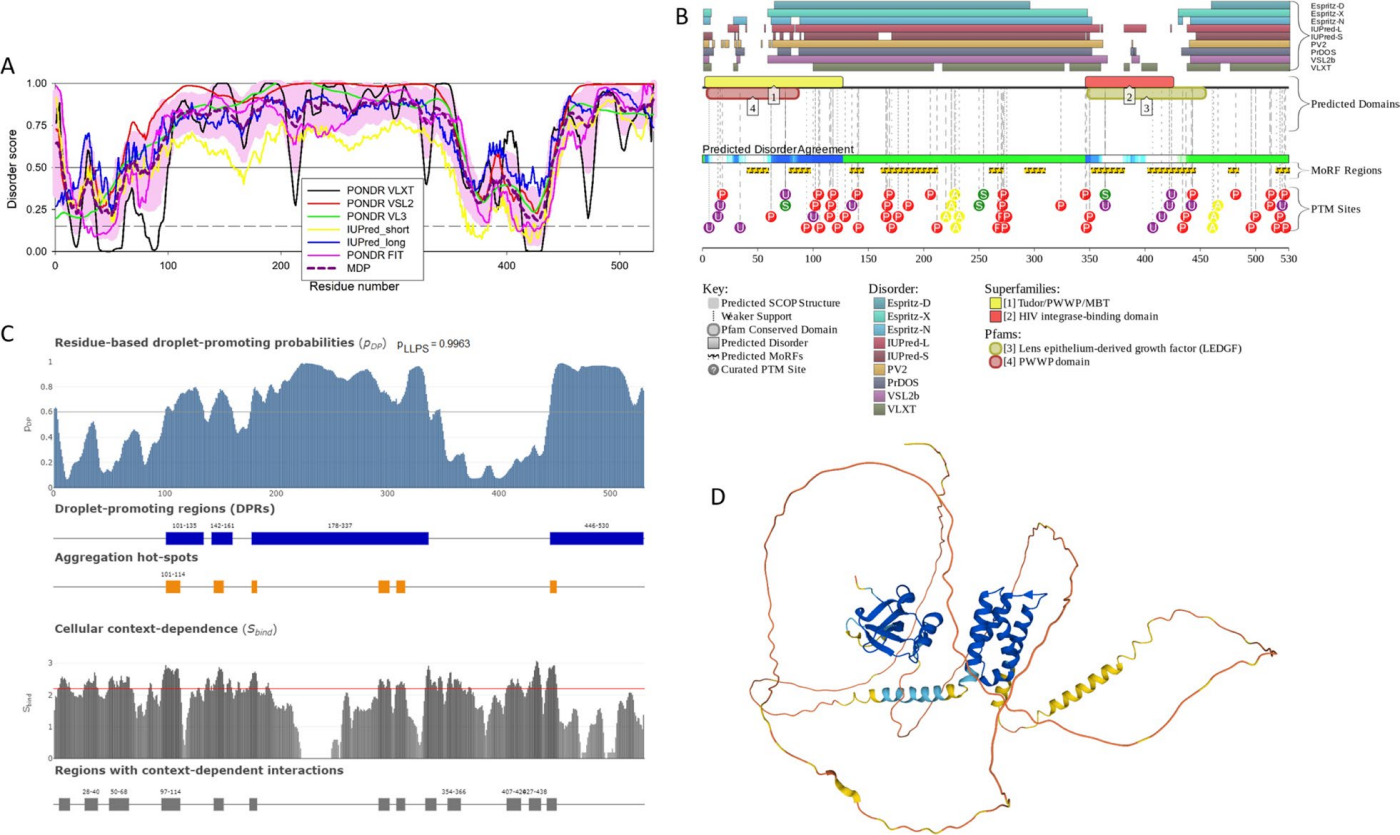
Functional disorder analysis of human LEDGF/p75 protein (UniProt ID: O75475). **A** Per-residue disorder profile generated by RIDAO [138]. **B** Functional disorder profile generated by the D^2^P^2^ platform [139]. Here, phosphorylation, ubiquitylation, sumoylation, and acetylation sites are shown by red, violet, green and yellow circles with characters P, U, S, and A, respectively. An interactive profile generated for this protein by D^2^P^2^ is available at https://d2p2.pro/view/sequence/up/O75475. **C** Evaluation of LLPS predisposition and existence of regions with context-dependent disorder [145]. **D** Model of the LEDGF/p75 structure generated by AlphaFold [147]

**Fig. 7:**
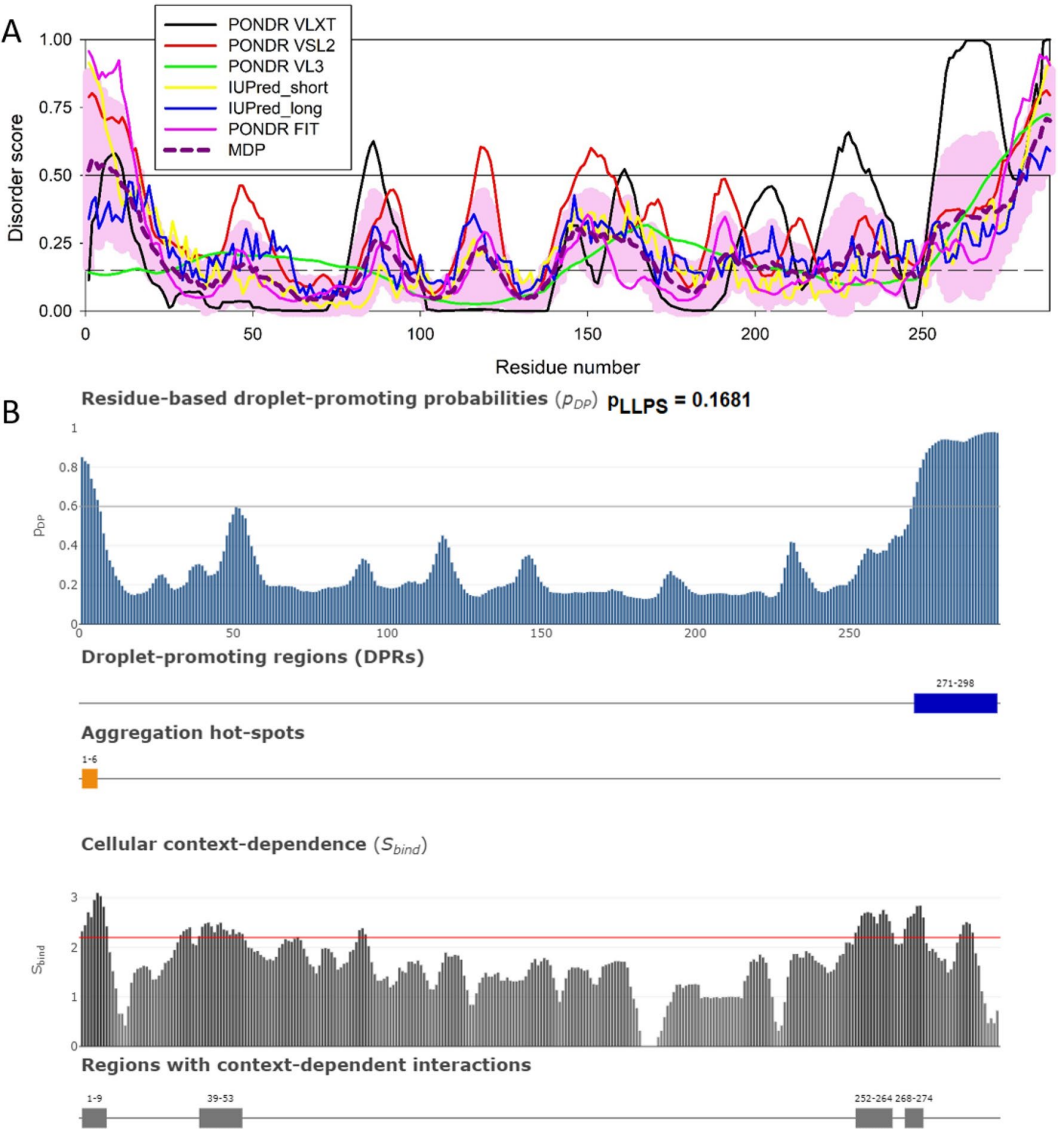
Disorder analysis of the HIV-1 integrase IN protein (UniProt ID: Q79666; residues 1149-1446). **A**. Per-residue disorder profile generated by RIDAO [122]. **B**. Evaluation of LLPS predisposition, the presence of droplet-promoting regions (DPRs), and existence of regions with context-dependent disorder [129]

HIV-MLOs also act as sites to pilot the HIV-1 genome in active chromatin regions. All around HIV-1 MLOs there is active chromatin similarly to the open chromatin that surrounds NSs. Active proviruses have been found to mainly localize in lens epithelium–derived growth factor (LEDGF) enriched chromatin regions near HIV-1 MLOs. LEDGF appears as clusters in the nucleus in macrophage-like cells and they are mainly distributed near HIV-1 MLOs when cells are infected [75]. This is in line with the calculated P_LLPS_ value ~ 1 (Fig. 6 and Table 1). Contrary to LEDGF, IN clusters are only visible inside HIV-1-MLOs but due to their low tendency to form condensates (IN p_LLPS_ value ~ 0.1), (Fig. 7 and Table 1) they likely serve as MLO clients [92] condensed within the HIV-MLO cores, while they are not associated with LEDGF when observed by imaging [75]. Indeed, highly active chromatin regions (type A compartment) are physically associated with NSs [93, 94], while repressive chromatin regions (type B compartment) associate with the nuclear periphery or the nucleolus [95, 96]. Importantly, HIV-1 proviruses have been found in speckle-associated domains (SPADs) by genomic [97] and nuclear spatial imaging analysis [75]. In line with these findings, it is known that active proviruses are excluded from the type B chromatin compartment, indeed HIV-1 integration in the human genome favours active genes and local hotspots [98]. A recent study shows that LLPS plays a key role in selecting HIV-1 hotspots to ensure efficient viral replication [75].

### Late replication steps in HIV-1

#### Transcription and proviral DNA reactivation

The engagement of *cis*-acting transcription factors with their cognate sequences in promoter sequences has taken on a new understanding. While transcriptional initiation generally involves several sequential events, it now is understood to involve inhibitory and stimulatory complexes [6, 99–101]. The late steps of retroviral replication involve proviral gene transcription driven by the long-terminal repeat (LTR) that harbours multiple *cis*-acting sequences and responsive to in large part host transcription factors. Several recent indicators show that proviral DNA transcription from latently infected cells also relies on phase separation of multi-component complexes. LLPS is also a key factor in the reactivation of HIV-1 proviral DNA transcription reactivation from latency, implicating components of the host histone chaperone chromatin assembly factor 1 and polycomb repressive complex 1 at the HIV-1 promoter [102]. Interestingly, phase separating fused-in-sarcoma and transcription super elongation complex component, AF4/FMR2 family member 4 (AFF4), both known to act via phase separation [103], act to suppress HIV-1 reactivation [104]. Few details exist on the precise involvement of condensates in HIV-1 gene transcription in actively replication cells, but in light of work in other systems that show the engagement of RNA Pol II processivity via positive transcription factor b (pTEFb) and AFF4 condensation [103] is suggestive of a role in HIV-1 DNA transcription. Co-transcriptional retroviral RNA condensates have not been identified except that there is some visible evidence of Rev-responsive element (RRE)-containing RNA-CRM1-Rev complexes that are able to recover following photobleaching using fluorescence recovery after photobleaching techniques [105] and recent work shows that Gag-containing unspliced RNA complexes are found in the cell nucleus [106]. These condensate-like ribonucleoproteins make their way into the cytoplasm through the nuclear pore complex, which also represents a liquid barrier compartmentalizing the cytoplasm from the nucleoplasm [107], as described above. Interestingly, studies on unrelated viruses show that cellular stress provokes activation of persistent viral infection mediated by biomolecular condensates in the host cytoplasm. It has been recently reported that phosphoprotein of mumps virus coincides with the phase separation of viral polymerase into pre-formed biomolecular condensates and the formation of a stable replication machinery [108]. Future work should reveal additional means by which retroviruses assemble condensates via LLPS for their nuclear intermediate replication steps.

#### Assembly, maturation and budding

**Fig. 8:**
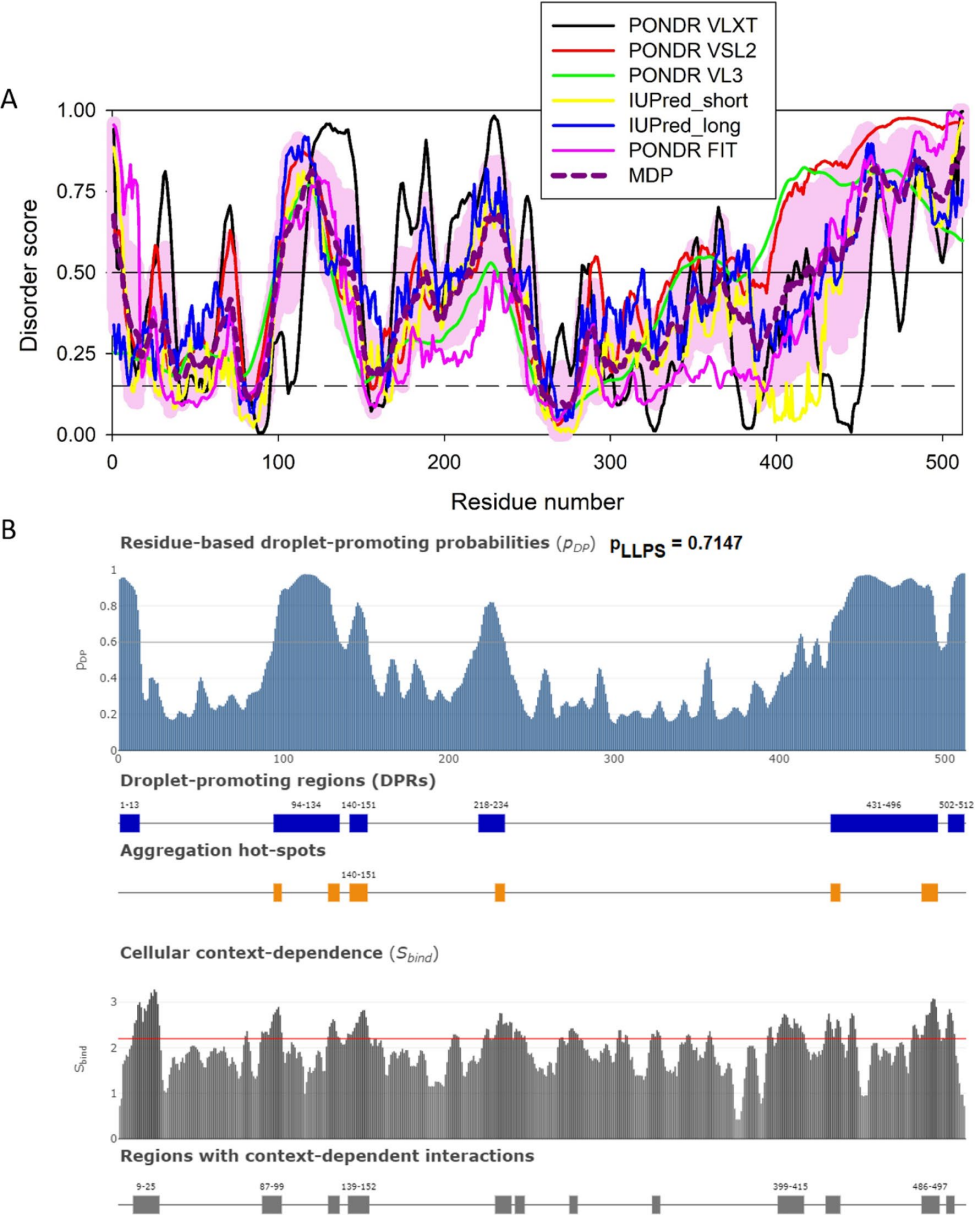
Disorder analysis of the HIV-1 precursor 55 kDa Gag protein (UniProt ID: P12493). **A** Per-residue disorder profile generated by RIDAO [138]. **B** Evaluation of LLPS predisposition and existence of regions with context-dependent disorder [145]

Classically, following nuclear export, the viral mRNAs are translated to viral structural, polymerase, accessory (auxiliary), and regulatory gene products for final assembly of virus particles. Depending on cell type, these components need to traffic to sites of assembly on membranes and assemble in a stochiometric manner. Few details are understood about the implication of phase separation in the assembly of these proteins; nevertheless bioinformatic analyses have revealed a propensity of viral proteins to phase separate and form phase separated droplets [109]. Full-length, precursor 55 kDa Gag (pr55Gag) on its own displays several intrinsically disordered regions that experimentally, is reflected in droplet formation in vitro [53] (Fig. 8 and Table 1), that bear characteristics of phase separated compartments such that they fuse and fission on coverslips or in the case of Rous sarcoma virus, form liquid phase, mobile droplets in the nucleus [110, 111].

**Fig. 9:**
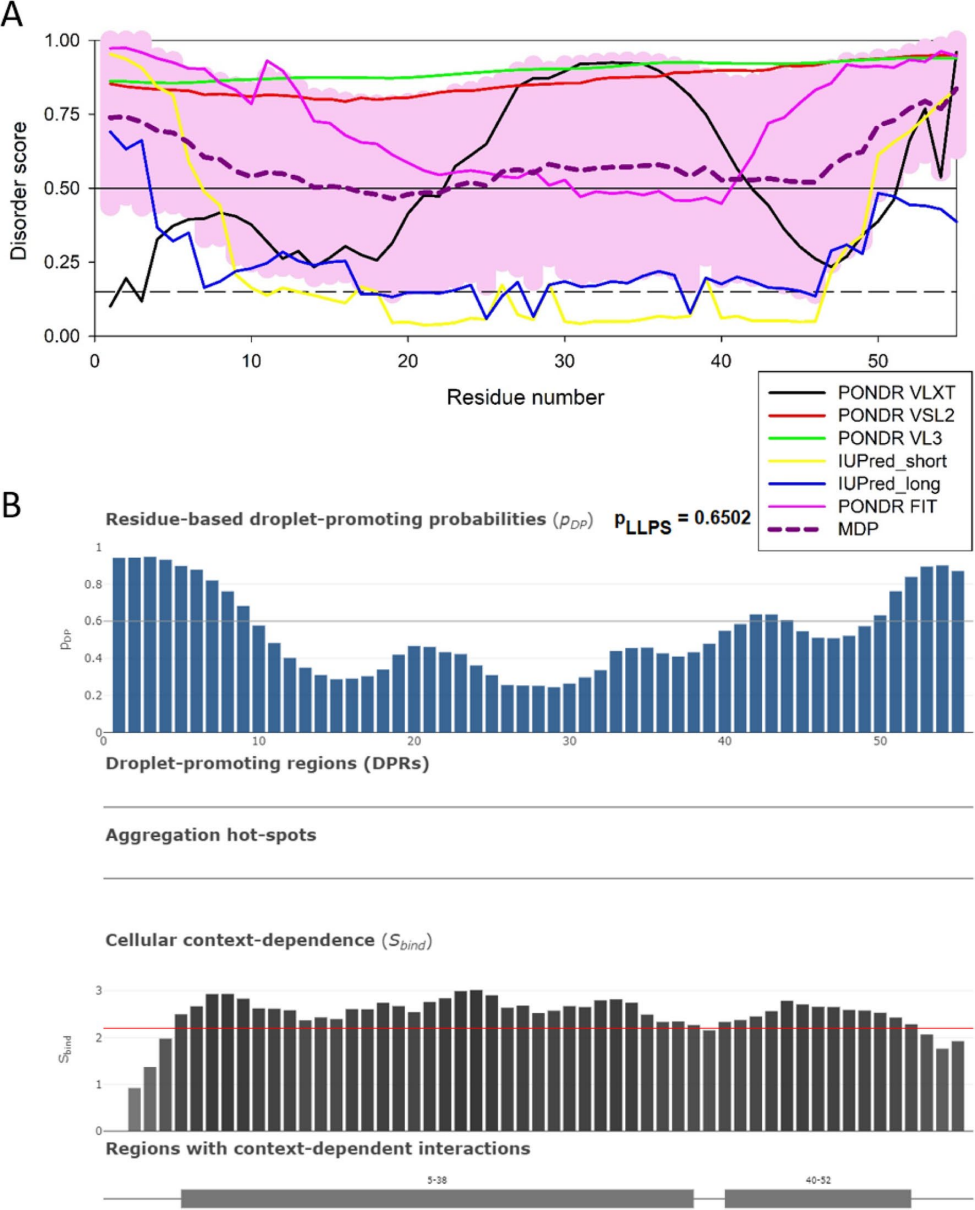
Disorder analysis of the HIV-1 nucleocapsid NC protein (UniProt ID: P12497; residues 378–432). **A** Per-residue disorder profile generated by RIDAO [138]. **B** Evaluation of LLPS predisposition and existence of regions with context-dependent disorder [145]

Later during virus assembly, as Gag recruits ESCRT complexes to induce membrane curvature, the viral protease auto-activates and begins processing the viral precursor proteins pr55Gag and pr160Gag/Pol during virus maturation. Interestingly, the addition of recombinant proteins making up a viral core (NC, IN, RT, vRNA, CA) co-assemble to attain a geometry characteristic of a *bona fide* core [92]. Processing of pr55Gag in cells with a recombinant and active viral protease resulted in abundant mobile, condensates in cells [92]. While NC was shown to co-assemble PR-containing condensates late at the end of budding, in elegant work [112], NC condensates exhibited strong “scaffolding” activity in this context (Fig. 9 and Table 1), with the core proteins RT, IN, PR (and the viral genomic RNA) as clients, relying on the scaffolding behaviour of HIV-1 NC in cells [92] consistent with work from the Lyonnais and Mirambeau labs [112] and predicted high capacity to form droplets (p_LLPS_ = 0.6502, Fig. 9B and Table 1). In this particular case, bioinformatic predictions are reflected by in vitro observations made in cells [92].

## What’s a virus without its host? Intrinsic disorder and LLPS propensities of host factors

**Fig. 10:**
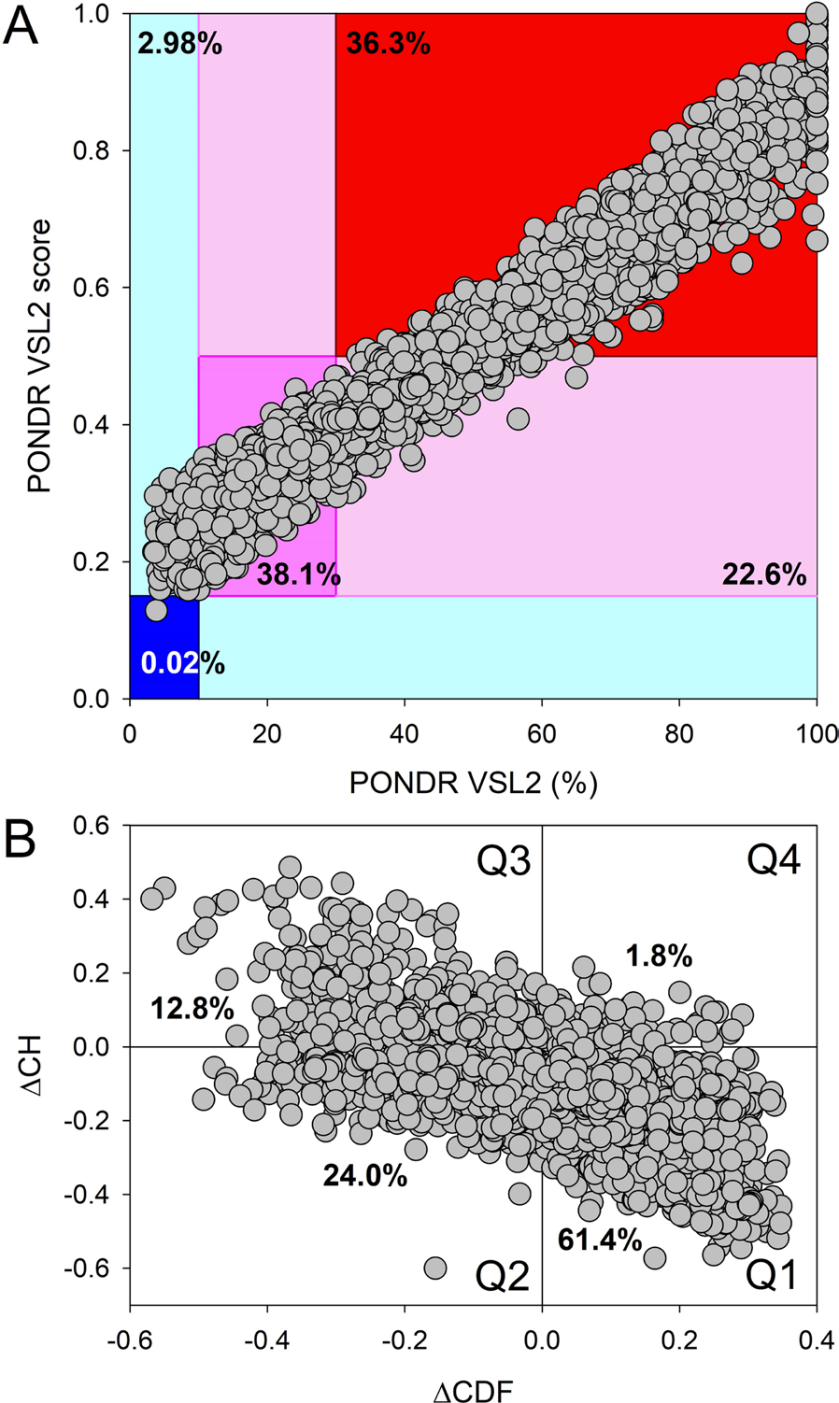
Overall disorder analysis of 4792 human proteins interacting with all HIV-1 proteins. Information about these host proteins was retrieved from the HIV-1 Interaction Database accessible at the National Institutes of Health: National Center for Biotechnology Information (see https://www.ncbi.nlm.nih.gov/genome/viruses/retroviruses/hiv-1/interactions/). **A** PONDR^®^ VSL2 mean disorder score vs. PONDR^®^ VSL2 (%) plot. Here, each point corresponds to a query protein, coordinates of which are evaluated from the corresponding PONDR^®^ VSL2 data as its mean disorder score (MDS) and percent of the predicted intrinsically disordered residues (PPIDR). MDS was calculated for each query protein as a protein length-normalized sum of all the per-residue disorder scores, whereas PPIDR was calculated as a protein length-normalized number of residues with the disorder scores of at least 0.5 multiplied by 100%. Color blocks are used to visualize proteins based on the accepted classification, with red, pink/light pink, and blue/light blue regions containing highly disordered, moderately disordered, and ordered proteins, respectively. Classification is based on the accepted practice, where a PPIDR value of less than 10% is taken to correspond to a highly ordered protein, PPIDR between 10 and 30% is ascribed to moderately disordered protein, and PPIDR greater than 30% corresponds to a highly disordered protein [148, 149]. Proteins can also be grouped based on their corresponding MDS values, being classified as highly ordered (MDS < 0.15), moderately disordered of flexible (MDS between 0.15 and 0.5) and highly disordered (MDS ≥ 0.5). Dark blue or pink regions correspond to the regions, where PPIDR agrees with MDS, whereas areas in which only one of these criteria applies are shown by light blue or light pink. Numbers within these segments reflect their protein content. **B** CH-CDF plot, where coordinates for a protein are calculated as the average distance of its CDF curve from the CDF boundary (X axis) and its distance from the CH boundary. Protein classification is based on the quadrant, where it is located: Q1, protein predicted to be ordered by both predictors. Q2, protein predicted to be ordered to by CH-plot and disordered by CDF. Q3, protein predicted to be disordered by both predictors. Q4, protein predicted to be disordered by CH-plot and ordered by CDF. CH-CDF analysis combines results from the charge-hydropathy (CH) and cumulative distribution function (CDF) plots, which are both binary predictors of disorder. For CH, net charge is plotted versus hydropathy for each protein [150]. Due to the observation that disordered proteins tend to have high net charge and low hydropathy, disordered and ordered proteins cluster two regions of the plot. A linear boundary separates disordered and ordered proteins [150, 151]. Proteins that are disordered appear above the boundary while ordered proteins appear below [150, 151]. In the CDF-plot predictor, PONDR scores for each residue of a single protein is plotted against their frequency within the sequence. If a CDF curve of a given protein is below the order–disorder boundary, this protein is considered disordered, and protein is ordered if the CDF curve is located above this boundary [151]. Data generated by CH- and CDF-plots are then combined to generate ΔCH-ΔCDF plot, which enables rapid discrimination between flavors of disorder [152, 153]. To this end, for each query protein, ΔCH, the vertical distance of the corresponding point in CH plot from the boundary, is calculated, whereas ΔCDF is computed as the average distance between the order–disorder boundary and the CDF curve. Then, ΔCH is plotted against ΔCDF resulting in a CH-CDF plot. Proteins in the top-left quadrant are predicted to be disordered by both CH and CDF, the ones in the bottom-left are predicted to be ordered by CH and disordered by CDF, the ones in the top-right are predicted to be disordered by CH and ordered by CDF, and in the bottom-right quadrant predicted to be ordered by both [152, 153]

Analysis of the intrinsic disorder propensity and LLPS potential of the 4,792 human proteins interacting with HIV-1 proteins, revealed that a very significant part of these HIV-1-interacting partners (> 36%) is predicted to be highly disordered, with even more of these proteins predicted to be moderately disordered (Fig. 10). Although these values are rather comparable to those found for the entire human proteome (e.g., see [113]), the HIV-1 interactome contains significantly less highly ordered proteins (0.02% vs. 0.04% and 2.98% vs. 5.1% in the dark-blue and light-blue segments of HIV-1 interactors and whole human genome, respectively), suggesting that the functionality associated with intrinsic disorder might be important for these host proteins. Table 2 represents the results of the evaluation of global intrinsic disorder predisposition of human proteins from several datasets related to the HIV-1 infection. Analyzed datasets include whole human proteome (20,317 proteins; https://www.uniprot.org/proteomes/UP000005640, accessed on 09.24.2022), 4792 human proteins interacting with HIV-1, 252 CA interactors, 221 GagPol interactors, 210 Integrase interactors, 657 Pr44Gag interactors, 106 Integrase interactors, 1667 Tat interactors, and 657 Vpr interactors. Information about human proteins in these datasets was retrieved from the HIV-1 Interaction Database accessible at the National Institutes of Health: National Center for Biotechnology Information (see https://www.ncbi.nlm.nih.gov/genome/viruses/retroviruses/hiv-1/interactions/, accessed on 12.01.2022).

**Table 2:** Evaluation of global intrinsic disorder predisposition of human proteins from several datasets related to HIV-1 infection

Dataset (number of proteins)	PONDR^®^ VSL2 analysis				CH-CDF analysis			
	Mostly ordered^a^	Moderately disordered^b^	Mostly disordered^c^	Highly disordered^d^	Q1	Q2	Q3	Q4
Whole proteome (20,317)	83(0.4%) 1030(5.1%)	6841(33.7%) 4267(21.0%)	8095(39.8%)	7381(36.3%)	12,014 (59.1%)	5177 (25.5%)	2501 (12.3%)	625 (3.1%)
All human interactors (4792)	1(0.02%) 143(2.98%)	1828(38.1%) 1082(22.6%)	1738(36.3%)	1534(32.0%)	2945 (61.4%)	1149 (24.0%)	612 (12.8%)	86 (1.8%)
Capsid interactors (252)	0(0.0%) 12(4.7%)	98(38.9%) 68(27.0%)	74(29.4%)	65(25.8%)	180 (71.4%)	49 (19.5%)	21 (8.3%)	2 (0.8%)
GagPol interactors (221)	0(0.0%) 6(2.7%)	99(44.8%) 32(14.5%)	84(38.0%)	75(33.9%)	122 (55.2%)	35 (15.8%)	59 (22.7%)	5 (2.3%)
Integrase interactors (210)	0(0.0%) 8(3.8%)	95(45.2%) 50(23.8%)	57(27.2%)	54(25.7%)	143 (68.1%)	37 (17.6%)	28 (13.3%)	2 (1.0%)
Pr44Gag interactors (657)	0(0.0%) 20(3.0%)	264(40.2%) 126(19.2%)	247(37.6%)	226(34.4%)	374 (56.9%)	140 (21.3%)	130 (19.8%)	13 (2.0%)
RT Interactors (106)	0(0.0%) 2(1.9%)	58(54.7%) 21(19.8%)	25(23.6%)	21(19.8%)	78 (73.6%)	19 (17.9%)	9 (8.5%)	0 (0.0%)
Tat Interactors (1667)	0(0.0%) 42(2.5%)	617(37.0%) 420(25.2%)	588(35.3%)	508(30.5%)	1034 (62.0%)	380 (22.8%)	234 (14.0%)	19 (1.2%)
Vpr interactors (657)	0(0.0%) 19(2.9%)	272(41.4%) 166(25.3%)	200(30.4%)	173(26.3%)	432 (65.8%)	140 (21.3%)	73 (11.1%)	12 (1.8%)

^a^Mostly ordered proteins are those found in the dark blue and light blue areas of the mean disorder score (MDS) vs. percent of predicted intrinsically disordered residues (PPIDR) plots; i.e., areas containing proteins with PPIDR < 10% and MDS < 0.15, where dark blue correspond to area where these two parameters agree (upper values in the corresponding cells), whereas light blue color show areas in which only one of these criteria applies (bottom values in the corresponding cells)

^b^Moderately disordered proteins are those found in the dark pink and light pink areas of the mean disorder score (MDS) vs. percent of predicted intrinsically disordered residues (PPIDR) plots; i.e., areas containing proteins with 10% ≤ PPIDR < 30% and 0.15 ≤ MDS < 0.1, where dark pink correspond to the area where these two parameters agree (upper values in the corresponding cells), whereas light pink color show areas, in which only one of these criteria applies (bottom values in the corresponding cells)

^c^Mostly disordered proteins are those found in the red area of the mean disorder score (MDS) vs. percent of predicted intrinsically disordered residues (PPIDR) plots; i.e., area containing proteins with PPIDR ≥ 30% and MDS ≥ 0.5

^d^Highly disordered proteins are a subset of mostly disordered proteins that are characterized by PPIDR ≥ 50% and MDS ≥ 0.5

**Fig. 11:**
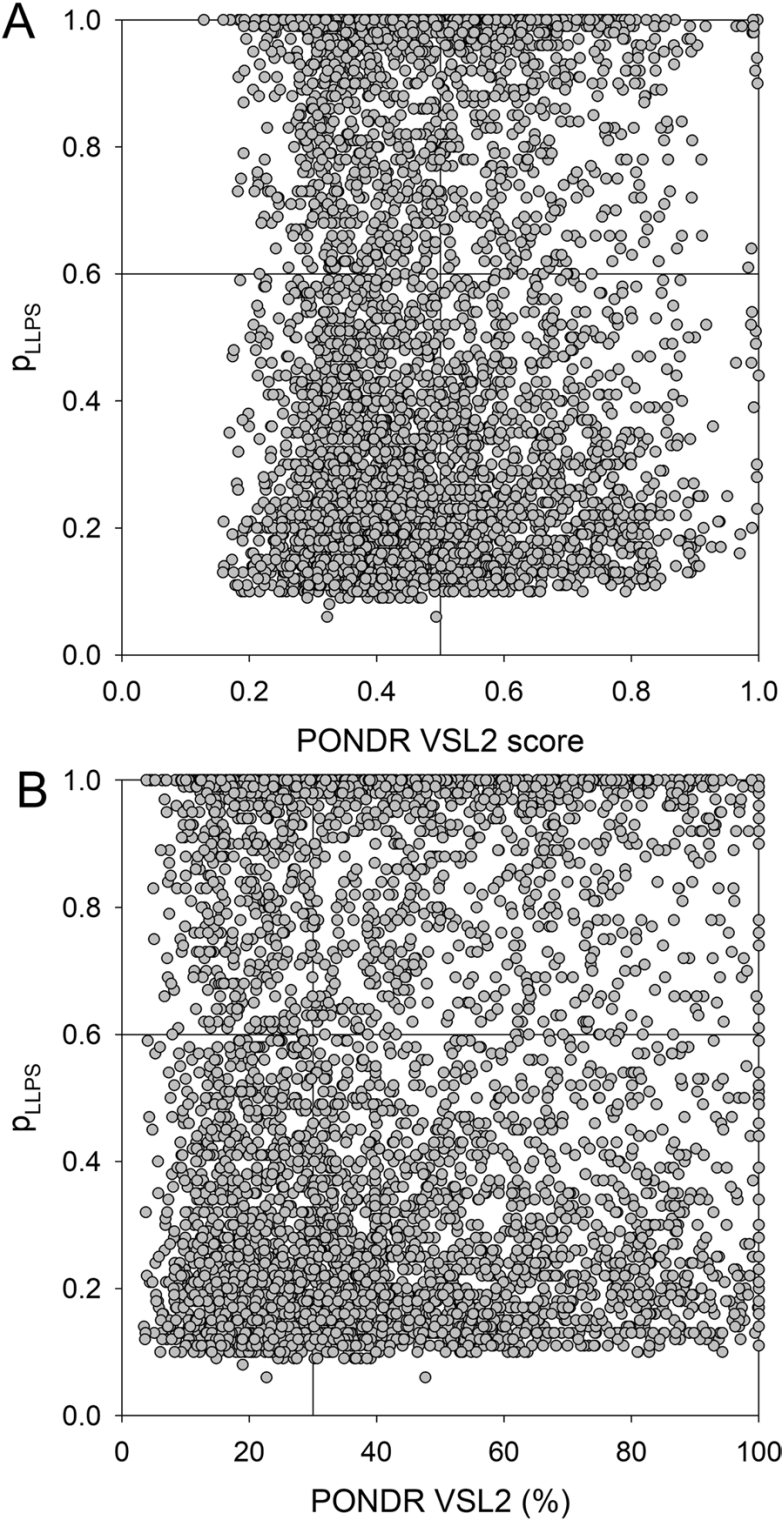
Intrinsic disorder and phase separation propensity of 4792 human proteins interacting with all HIV-1 proteins. **A** p_LLPS_ vs. PONDR^®^ VSL2 disorder score dependence. In these analyses, proteins with p_LLPS_ ≥ 0.6 are expected to serve as LLPS drivers, whereas proteins with the disorder scores ≥ 0.5 are predicted as mostly disordered. Among the 4792 human proteins interacting with all HIV-1 proteins, 1738 proteins (36.27%) are expected to be mostly disordered, and 1669 (34.83%) are expected to serve as LLPS drivers. Among the LLPS drivers, 655 proteins (39.24%) are mostly disordered, indicating that 13.67% of HIV-1-interactors are mostly disordered proteins driving LLPS. **B** Dependence of the p_LLPS_ on the percent of disordered residues predicted by PONDR^®^ VSL2. Proteins with p_LLPS_ ≥ 0.6 are expected to serve as LLPS drivers, whereas proteins with the PPIDR ≥ 30% are predicted as highly disordered. Based on the outputs of these analyses, 2820 human proteins from HIV-1 interactome (58.84%) are expected to be highly disordered. Among 1669 of LLPS drivers, 1038 proteins (62.19%) are expected to be highly disordered, indicating that 21.66% of HIV-1-interactors are highly disordered LLPS drivers

As it was already emphasized, one of the crucial functions of intrinsically disordered proteins and regions is their involvement in LLPS and associated biogenesis of MLOs [16, 18, 19, 114, 115]. Figure 11 shows that 1670 human proteins interacting with HIV-1 (34.8%) are predicted to have propensity for phase separation higher than the 0.6 threshold, and therefore are expected to serve as scaffolds or drivers of phase separation. About 40% of these LLPS drivers are predicted to be highly disordered (their mean disorder scores exceed the threshold of 0.5), and most of them (1037 or 62%) are expected to contain more than 30% of disordered residues. Most of the LLPS drivers contain DPRs (in fact, only 14 of these proteins or 0.8% do not have DPRs), with number of DPRs can be as high as 48 (84 LLPS drivers have at least 10 DPRs). More than half of the remaining 3122 HIV-1-interactors (1674 or 53.5%) were predicted do not have any DPRs, whereas 1448 proteins from this dataset (or 30.2% of the HIV-1 interactome) are predicted to have 1 to 5 DPRs and therefore are expected to serve as droplet clients. In other words, more than 65% of HIV-1-interactors are expected to be engaged in LLPS. On the other hand, in the entire human proteome containing 20,367 proteins), 7570 and 5252 proteins (37.2% and 25.8%) are expected to act as LLPS drivers and droplet clients, respectively. Therefore, the HIV-1 interactome contains less LLPS drivers and more droplet clients than those found in the human proteome. Curiously, 1448 droplet clients found in HIV-1 interactome are expected to be more disordered than 1674 HIV-1 interactors not involved in droplet biogenesis. This prediction is in agreement with the comparison between IN and CPSF6 clusters in the infected cells for their ability to form condensates. BMCs are highly dynamic, and this feature can be measured by fluorescence recovery after photobleaching. Results clearly show that IN proteins are unable to recover after photobleaching, contrary to CPSF6 proteins that show high mobility giving rise to the restoration of the fluorescent droplet after photobleaching [75].

## Therapeutics and perspectives of targeting MLOs

Biomolecular condensates formed during virus replication or those formed aberrantly in other human diseases represent new and emerging targets for therapeutic intervention [116–118] (for recent reviews on phase separation and viruses, please see: [34, 39, 119–121]). Further understanding of LLPS in retroviral replication will lay the foundation for future study of retroviral MLOs that exhibit fluid-like behaviour during replication. Long-term goals in LLPS virology research include the screening for compounds that modulate the stability of viral condensates [122–126], such as those identified to rigidify respiratory syncytial virus BMCs [127] or those that target scaffolding SARS-CoV-2 N condensates to evade immune signalling [128–130] or finding mutants in HIV-1 core proteins that lead to aberrant condensation such as those found to be etiologic for human disease [118, 131]. Based on our current understanding of HIV-1 biology for example, it follows that the HIV-1 BMC makes its way through a nuclear pore condensate and then into a nuclear condensate, the nuclear speckle to complete reverse transcription [90] (Fig. 1). Integration is then achieved with the help of LEDGF/p75 [132, 133] that form condensates near HIV-1 MLOs in macrophage-like cells [75]. LEDGF is a host protein that possesses intrinsically disordered domains to form clusters (Fig. 6). While several retroviral NC proteins form droplets in vitro [53], it remains to be determined if condensation through LLPS plays a fundamental role in other retroviruses. Future perspectives for HIV-1 will include the study on how condensation plays roles in early pre-integration, integration steps [34, 75, 90] as well late in HIV-1 replication in transcription, considering that recently characterized BMCs involving host histone chaperone chromatin assembly factor 1 and polycomb repressive complex 1 and their influence on HIV-1 reactivation from latency, acting near the HIV-1 promoter [102, 134]. Targeting viruses with BMC therapeutics will be multi-pronged since virus infection, including HIV-1, exacerbates the outcomes of neurodegenerative diseases that are often dependent on aberrant BMC assemblies [135, 136].

## Conclusions

Ultimately, the characterization of biomolecular condensates in many viruses, and not just retroviruses, have revealed mechanistic ways in which viruses mimic, highjack or usurp host membraneless organelles. In retroviruses, many of the bioinformatics analyses on disorder and whether viral proteins are drivers/scaffold or client of biomolecular condensates shown here are reflected by recent experimental observations. On the other hand, many of the proposed roles for condensates proposed here await confirmation by further in vitro and in cell assays, and biophysical analyses including roles for condensation through phase separation during viral assembly on membranes, during nuclear entry of core components and early and late nuclear events. The plasticity of BMCs should seamlessly orchestrate various replication steps (Fig. 1), especially enzymatic activities (e.g., PR, RT, IN) [112] and may allow for virus-host assemblies and interactions through engagement and disengagement [137] that are obligatory throughout the replication cycles of retroviruses and other viruses.

## Data Availability

Not applicable.

## Abbreviations

AFF4: AF4/FMR2 family member 4
BMCs: Biomolecular condensates
CA: Capsid protein
COVID-19: Coronavirus disease-2019
CPSF6: Cleavage and polyadenylation specific factor 6
CRM1: Chromosomal maintenance 1 (or Exportin 1)
DNA: Deoxyribonucleic acid
DPR: Droplet-promoting region
Env: Envelope protein
FG: Phenylalanine-glycine
G3BP1: Ras GTPase-activating protein-binding protein 1
Gag: Group specific antigen or Gag polyprotein
HIV-1: Human immunodeficiency virus-type 1
IDP: Intrinsically disordered protein
IDR: Intrinsically disordered region
IN: Integrase protein
LAD: Lamin associated domain
LEDGF/p75: Lens epithelium-derived growth factor
lncRNA: Long non-coding RNA
LLPS: Liquid–liquid phase separation
LTR: Long-terminal repeat
MCD: Mixed charge domain
MDS: Mean disorder score
MLO: Membraneless organelles
MoRF: Molecular recognition feature
N: Nucleocapsid (SARS-CoV-2)
NC: Nucleocapsid protein (HIV-1)
NPC: Nuclear pore complex
NSs: Nuclear speckles
Nup: Nucleoporin
Nup153: Nucleoporin 153
p_LLPS_: Probability of liquid–liquid phase separation
pr55Gag: Precursor 55 kDa Gag
PONDR: Predictor of natural disordered regions
PPIDR: Predicted intrinsically disordered residues
pTEFb: Positive transcription elongation factor b
PTM: Posttranslational modification
RBD: RNA-binding domain
RNA: Ribonucleic acid
RRE: Rev-responsive element
RRM: RNA-binding motif
rRNA: Ribosomal RNA
RT: Reverse transcriptase enzyme
SARS-CoV-2: Severe acute respiratory syndrome coronavirus 2
SPAD: Speckle-associated domain
vRNA: Viral RNA
ZnF: Zinc finger domain
